# Helios Should Not Be Cited as a Marker of Human Thymus-Derived Tregs. Commentary: Helios^+^ and Helios^−^ Cells Coexist within the Natural FOXP3^+^ T Regulatory Cell Subset in Humans

**DOI:** 10.3389/fimmu.2016.00276

**Published:** 2016-07-08

**Authors:** Eyad Elkord

**Affiliations:** ^1^Cancer Center, Qatar Biomedical Research Institute, College of Science and Engineering, Hamad Bin Khalifa University, Qatar Foundation, Doha, Qatar; ^2^College of Medicine and Health Sciences, United Arab Emirates University, Al Ain, United Arab Emirates; ^3^Biomedical Research Center, School of Environment and Life Sciences, University of Salford, Salford, UK; ^4^Institute of Cancer Sciences, University of Manchester, Manchester, UK

**Keywords:** T regulatory cells, Foxp3, Helios, tTregs, pTregs

Thymus-derived Tregs (tTregs) mediate peripheral tolerance, which benefits the host by controlling inflammation and preventing autoimmunity, whereas peripherally induced Tregs (pTregs) mediate tumor-induced suppression, which hurts the host by suppressing beneficial anti-tumor immunity (1–4). In 2010, Dr. Shevach’s group reported that Helios expression discriminates tTregs from pTregs (5). This work generated a lot of excitement due to the real need for markers to discriminate the good tTregs from the bad pTregs. This is of particular importance to specifically target pTregs to enhance anti-tumor immunity, while reserving tTregs to avoid provoking autoimmune diseases. Soon after, this work was questioned, and several studies showed that Helios can be induced in Foxp3^+^ T cells (6). A great wealth of recent studies excludes the value of Helios as a marker of tTregs. For example, a strong Helios expression can be induced in pTregs (7), and Helios expression is a marker of T cell activation and proliferation (8). Furthermore, it was confirmed that both Helios^+/−^ subsets coexist within human FoxP3^+^ tTreg (9). A more recent study showed that neither Helios nor Neuropilin-1 expressions could identify Tregs of thymic or peripheral origin (10).

Regarding Helios function, Helios regulates Treg functional stability by inducing epigenetic silencing of IL-2 expression, and loss of Helios expression in Tregs enhanced expression of the IL-2 gene resulting in increased Treg proliferation and secretion of IL-2 following activation, as well as impaired suppressive activity (11). A more recent study by Dr. Shevach’s group reported that Helios controls some aspects of Treg-suppressive function (12). Interestingly, impairing Helios expression in Foxp3^+^ Tregs results in defective Tregs, and Helios is required for their stable inhibitory activity (13). Additionally, we found that Helios, and not FoxP3, is the marker of activated Tregs expressing immunosuppressive markers GARP/LAP (14).

Taken together, Helios expression confers stable phenotype of Tregs, and FoxP3^+^Helios^+^ Tregs have enhanced immunosuppressive characteristics, compared with FoxP3^+^Helios^−^ Tregs (8, 15). Despite all these several recent studies confirmed that Helios is not a tTreg marker, unfortunately many studies cited/are still citing Helios as a tTreg marker. I believe that it is of great importance to make this very clear to avoid further confusion to the scientific community and not to cite Helios as a marker of tTregs anymore.

## Author Contributions

The author conceived the idea and wrote the manuscript.

## Conflict of Interest Statement

The author declares that the research was conducted in the absence of any commercial or financial relationships that could be construed as a potential conflict of interest.
